# Effect of Collaborative Review of Electronic Patient-Reported Outcomes for Shared Reporting in Breast Cancer Patients: Descriptive Comparative Study

**DOI:** 10.2196/26950

**Published:** 2021-03-17

**Authors:** Andreas Trojan, Basil Bättig, Meinrad Mannhart, Burkhardt Seifert, Mathis N Brauchbar, Marco Egbring

**Affiliations:** 1 OnkoZentrum Zürich Zürich Switzerland; 2 Onkologie Bellevue Zürich Switzerland; 3 Onko-hämatologisches Zentrum Zug Switzerland; 4 Epidemiology, Biostatistics and Prevention Institute University of Zurich Zürich Switzerland; 5 Mobile Health AG Zürich Switzerland; 6 ePha Zürich Zürich Switzerland

**Keywords:** cancer, Consilium Care, smartphone app, eHealth, electronic patient-reported outcomes, Common Terminology Criteria for Adverse Events

## Abstract

**Background:**

Digital monitoring of treatment-related symptoms and self-reported patient outcomes is important for the quality of care among cancer patients. As mobile devices are ubiquitous nowadays, the collection of electronic patient-reported outcomes (ePROs) is gaining momentum. So far, data are lacking on the modalities that contribute to the quantity and quality of ePROs.

**Objective:**

The objective of our study was to compare the utilization of two versions of a subsequently employed mobile app for electronic monitoring of PROs and to test our hypothesis that a shared review of symptoms in patient-physician collaboration has an impact on the number of data entries.

**Methods:**

The Consilium Care app engages cancer patients to standardize reporting of well-being and treatment-related symptoms in outpatient settings. For descriptive comparison of the utilization of two slightly different app versions, data were obtained from an early breast cancer trial (version 1 of the app, n=86) and an ongoing study including patients with advanced disease (version 2 of the app, n=106). In both app versions, patients and doctors were allowed to share the information from data entries during consultations. Version 2 of the app, however, randomly selected symptoms that required a detailed and shared regular patient-doctor review in order to focus on the collection and appropriate interpretation regarding awareness and guidance for severity grading. The numbers and types of symptom entries, satisfaction with both app versions, and patients’ perceived effects during consultations were included for analysis.

**Results:**

Symptom severity grading was performed according to the Common Terminology Criteria for Adverse Events (CTCAE) using a horizontal slider and was indicated in descriptive terminology in both apps, while a graphical display facilitated the illustration of symptom history charts. In total, 192 patients electronically reported 11,437 data entries on well-being and 33,380 data entries on individual symptoms. Overall, 628 (of 872 intended) requested patient-doctor symptom reviews were performed in version 2 of the app. Both the amount of data entries per patient and day for well-being (version 1 vs version 2: 0.3 vs 1.0; *P*<.001) and symptoms (version 1 vs version 2: 1.3 vs 1.9; *P*=.04) appeared significantly increased in version 2 of the app. Overall satisfaction with both app versions was high, although version 2 of the app was perceived to be more helpful in general.

**Conclusions:**

Version 2 of the app showed much better results than version 1 of the app. A request for collaborative patient-doctor symptom review is likely to affect the number of digital symptom data entries. This app shows high potential to improve the patient-doctor experience.

**Trial Registration:**

ClinicalTrials.gov NCT02004496; https://clinicaltrials.gov/ct2/show/NCT02004496 and ClinicalTrials.gov NCT03578731; https://clinicaltrials.gov/ct2/show/NCT03578731

## Introduction

Despite the considerable progress of cancer treatment in recent decades, shortcomings still remain in patient self-management and communication with doctors. Most patients are motivated to spend time and effort in documenting their symptoms for shared reporting with physicians during consultations. However, the collection of electronic patient-reported outcomes (ePROs) is now becoming widespread, since mobile health solutions harbor the potential to improve symptom documentation regarding treatment pathways and facilitate communication between stakeholders [1,2]. To meet these requirements, mobile apps have been designed and tested with input from patients, nurses, and doctors and have gained attention with respect to improving efficacy and safety data in oncology trials and drug discovery studies [3-5]. The benefit of digital patient monitoring during immunotherapy in cancer has been demonstrated in terms of a more efficient symptom assessment and patient-doctor communication, as well as a decreased need for telephone consultations [6-8]. Integrating ePROs for symptom monitoring during routine cancer care has also been associated with increased survival due to early responsiveness to symptoms, longer tolerance, and continuation of chemotherapy, as well as a potential reduction in follow-up costs [9,10]. Recent studies have explored patient compliance rates, with the use of symptom alerts emphasizing the impact of structured graphical displays on outcome reporting [3,4]. Consequently, several digital platforms are now implementing ePROs that allow cancer patients to capture symptoms in a timely and structured manner and to share data with treatment teams. Some platforms also apply automatic algorithms, which indicate alert notifications to patients and treatment centers if symptoms worsen [2,11,12]. We previously reported on the efficacy of the Consilium Care mobile smartphone app in a randomized clinical trial demonstrating that its use could stabilize daily functional activity and well-being of breast cancer patients in collaboration with their physicians [1]. Currently, efforts using version 2 of the Consilium Care app are being made to demonstrate the reliability of electronically captured patient-reported symptom entries upon shared reporting with physicians in routine cancer care for the early detection of critical symptoms [13].

In this study, we describe and compare the functionality and utility of two consecutively developed and slightly different Consilium Care app versions for collecting ePROs and test our hypothesis that a requested review of symptoms in a patient-physician collaboration would impact the frequency or number of digital data entries.

## Methods

In order to compare the functionality and utility of both Consilium Care smartphone apps (designed and intended for clinical outcome research), we referred to a cohort of breast cancer patients receiving systemic therapy, demonstrated baseline characteristics, and indicated systemic treatment regimens. Version 1 of the app was previously used in a prospective randomized controlled trial (NCT02004496), while the recently modified version 2 of the app is still being applied in an observational study [1,13]. The observational trial cohort (version 2 of the app) was included in this comparison study since information on utility became available from a subset of breast cancer patients, while the greater part of the participants in this study were treated for cancer of the lung, colon, and prostate, and lymphoma. Eligible participants for both trials were recruited consecutively and without preselection. Recording of well-being and symptoms usually started on the day of the initiation or change of anticancer treatment and continued during an observational period of 6 weeks for version 1 of the app and 12 weeks for version 2 of the app.

Both versions of the Consilium Care app were developed to continuously record symptoms and treatment side effects in cancer patients according to the Common Terminology Criteria for Adverse Events (CTCAE) [10] but were not designed to send questionnaires to patients. Data entry displays for patients in both apps provided similar functions, although they were presented in a slightly different manner. Version 1 of the app collected data on the recording of symptoms, well-being, and activities of daily living. However, the concept for a presumably more modern and user-centered design of version 2 of the app presented a greater range of available symptoms and was implemented with the help of doctors, nurses, and patients.

**Figure 1:**
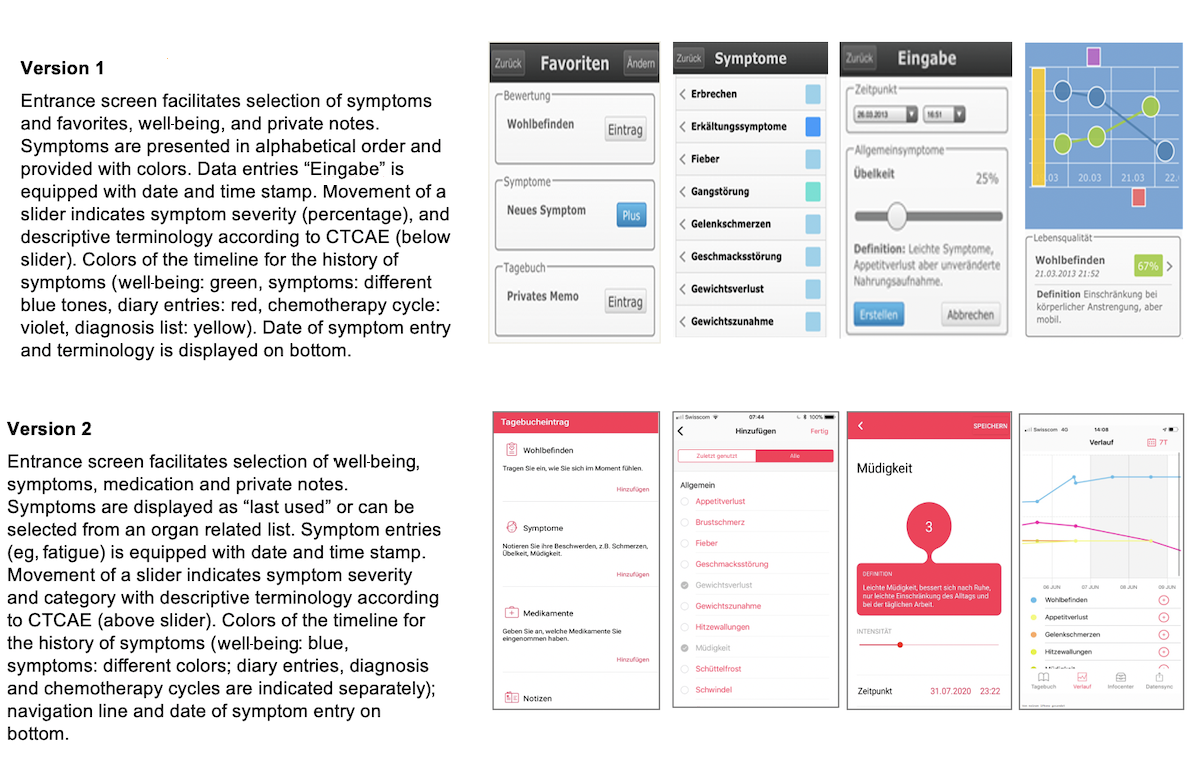
Different graphical displays of the two app versions. CTCAE: Common Terminology Criteria for Adverse Events.

Graphical displays for entering well-being, symptoms and corresponding grading, private notes, and medications, as well as the “time line” of the patient history of symptoms in both app versions are shown in Figure 1. A horizontal slider on a visual analog scale could be moved to indicate symptom severity and category according to the CTCAE, as displayed below (version 1 of the app) or above (version 2 of the app) the slider. Thirty symptoms were available to indicate severity, onset, and duration in version 1 of the app, and 52 symptoms were available for the same indications in version 2 of the app [1,8]. The first five categories were presented as a visual analog scale, while the sixth category, death, was omitted. Depending on the patient’s input, frequently reported symptoms were either displayed as “favorites” (version 1 of the app) or “last used” (version 2 of the app) (Figure 1).

Patients could also add private notes or additional symptoms and any medical measures undertaken as free text in version 1 of the app and in a more structured manner in version 2 of the app. In addition, patients indicated their daily functional activities according to the Eastern Cooperative Oncology Group (ECOG) performance status, and information for self-care (derived from the Swiss Cancer League) was displayed by the app depending on the severity of symptoms upon data entry (not shown). The history of recorded data was displayed automatically in both Consilium Care versions in the form of a graph (Figure 1) [4]. Patients were assigned to medical oncology visits every 3 weeks for shared reporting, which were preferentially scheduled on days of chemotherapeutic interventions. During consultation visits, nurses and doctors reminded the participants to use the app. If indicated, patients using version 2 of the app also received push notifications every 3 days to remind them of the need for data entry. Furthermore, at regular intervals, version 2 of the app randomly selected two patient-reported symptoms that were entered during the past 20 days. Patients and doctors were then prompted to perform a detailed and shared review of these symptoms, in order to focus on the collection and appropriate interpretation regarding awareness and guidance for symptom severity grading. Up to four such reviews with two symptoms each were planned according to the scheduled visits. At the end of each observational period, participants were asked to complete a questionnaire on paper reviewing the utility of the app and satisfaction with it in order to evaluate the quality of care and the relationship between the patient and physician during the course of treatment. The rating was completed after study participation using a 5-point Likert scale with scores ranging from 1 (agree not at all) to 5 (agree very strongly).

Both versions of the Consilium Care app were available on the most common platforms (Apple App Store or Google Play Store). After loading the app, a QR scanner was available to decode the patients’ personalized QR code. The participating centers were responsible for data entry into the electronic data capture (EDC) system. Patient data were stored in a designated ISO 27001-certified data center. ePRO data were synchronized between the smartphone app and the databank accordingly. For each patient’s convenience, a summary of diagnostic work-up, treatment medications, and the contact information of the respective treatment center were displayed on both app versions.

For descriptive analysis, categorical variables were presented as frequencies and percentages. Differences between groups were assessed using Pearson chi-square test. Age was presented as mean (SD). The numbers of entries per patient and day were not normally distributed and hence were reported as medians with IQR. The Mann-Whitney test was performed to compare groups. Two-sided *P* values ≤.05 were considered statistically significant. There was no adjustment for multiple testing. All statistical analyses were performed using R version 4.0.0 (R Foundation for Statistical Computing).

The research complies with the guidelines for human studies and was conducted ethically in accordance with the World Medical Association Declaration of Helsinki. We state that all participants provided written informed consent to publish their data. Both study protocols (NCT02004496 and NCT03578731) were approved by the local ethics committee on human research.

## Results

### Baseline Characteristics

**Figure 2:**
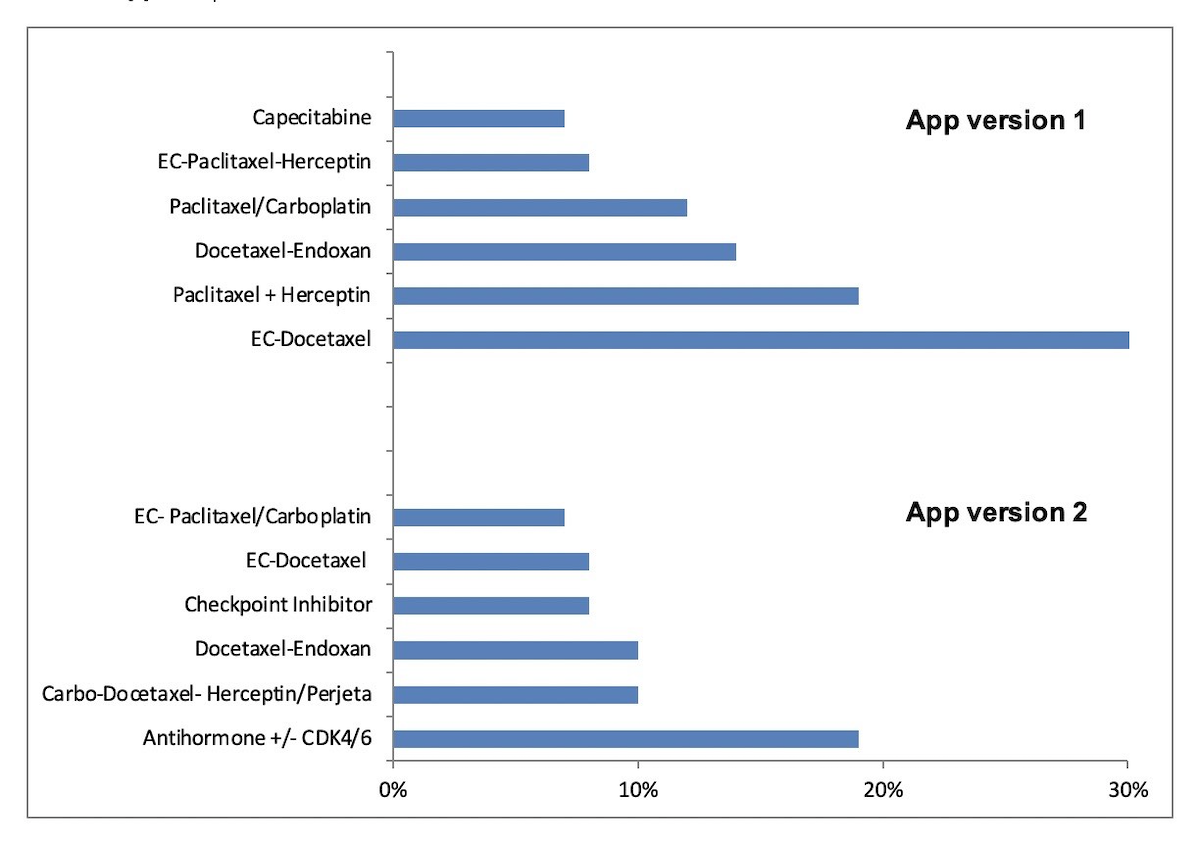
Frequency of the most commonly applied anticancer therapies during Consilium app use.

Between December 2013 and July 2015, 86 breast cancer patients using version 1 of the app completed all study visits, while for version 2 of the app, data from a subset of 106 patients were available for analysis upon recruitment from November 2018 to October 2019. For descriptive comparison, baseline characteristics as distributed between both patient groups are displayed in Table 1. The mean age of the patients using version 1 of the app was 52 years, and that of the patients using version 2 of the app was 56 years (Table 1). All 86 patients using version 1 of the app were treated for early stage disease, and two-thirds (n=54, 63%) of these patients were treated in an adjuvant setting. In contrast, about half (n=56, 53%) of the patients using version 2 of the app received treatment for advanced disease with noncurative intention. In patients using version 1 of the app, a total of seven distinct chemotherapeutic agents in six different chemotherapy regimens were administered (Figure 2), whereas a much greater variety of 16 distinct antitumoral agents, including antihormones, CDK4/6 inhibitors, and immunotherapies, were applied in patients using version 2 of the app. During the ePRO reporting period, the most frequent chemotherapy regimens applied in early stage breast cancer were epirubicin/cyclophosphamide (n=32), paclitaxel/ trastuzumab (n=19), and paclitaxel/carboplatin (n=12). In contrast, for users of version 2 of the app, the most commonly used therapeutic regimens were antihormones ± CDK4/6 inhibitors (n=25), carbo-docetaxel-Herceptin/Perjeta (n=13), docetaxel-endoxan (n=13), and checkpoint inhibitors (n=11) (Figure 2). Owing to more advanced disease stages and neoadjuvant regimens, CDK4/6 inhibitors and anti-HER2 antibodies were among the most applied drugs in the patient cohorts.

**Table 1:** Patient demographics.

Characteristic		Consilium Care app		*P* value
		Version 1 (n=86)	Version 2 (n=106)	
Age (years), mean (SD)		52 (11)	56 (12)	.002
Female, n (%)		85 (99%)	106 (100%)	0.45
Male, n (%)		1 (1%)	0 (0%)	
**Intention, n (%)**				
	Adjuvant	54 (63%)	34 (32%)	<.001
	Neoadjuvant	32 (37%)	16 (15%)	<.001
	Noncurative	0 (0%)	56 (53%)	<.001
	Breast cancer	86 (100%)	106 (100%)	1.0
**Well-being entries (total), n**		1430	10,007	
	Per patient	16	94	
	Per patient and day, median (IQR)	0.3 (0.02-0.8)	1.0 (0.8-1.2)	<.001
**Symptom entries (total), n**		9271	24,109	
	Per patient	107	227	
	Per patient and day, median (IQR)	1.3 (0.6-3.0)	1.9 (1.1-3.5)	.038

### Well-Being and Symptoms

Since neither of the Consilium Care versions was designed to send questionnaires, reporting on well-being and symptoms was primarily performed on the patient’s individual motivation, although push notifications were sent every 3 days if indicated. Overall, a high absolute amount of data entries on well-being and symptoms was captured in both versions of the app. Regarding well-being, a total of 11,437 data entries were reported, of which 1430 entries were derived from 86 patients using version 1 of the app during an observational period of 6 weeks (average of 41 days), while 10,007 data entries were derived from 106 patients using version 2 of the app during an observational period of 12 weeks (average of 91 days). Considering the time point of treatment (neo/adjuvant vs noncurative), we found that noncurative patients statistically entered more well-being data (median [IQR]: neo/adjuvant, 1.0 [IQR 0.5-1.1] and noncurative, 1.1 [IQR 0.9-1.4]; *P*<.001). However, both patient groups (curative and noncurative) using version 2 of the app reported their well-being more than twice as often as early stage breast cancer patients using version 1 of the app (version 1 vs version 2: 0.3 vs 1.0; *P*<.001) (Table 1). Since both app versions displayed the input control for well-being in a similar manner, this observation seemed unlikely to be associated with design features, but could be attributed to the effects of shared reporting.

In summary, all 192 patients generated a large absolute number (33,380) of electronically reported symptoms and side-effects (9271 in version 1 and 24,109 in version 2), suggesting easy use of control panels and sliders in both app versions. From the 106 patients using version 2 of the app, a total of 628 (of 872 intended) patient-doctor shared reviews were performed on randomly selected symptoms that had been entered during the previous 20 days of the respective period. Since the number of reported symptoms per patient and day appeared significantly higher in users of version 2 of the app (version 1 vs version 2: 1.3 vs 1.9; *P*=.038), the implementation of a request for shared symptom review was likely to have stimulated an increase in the frequency or number of symptom data entries.

**Figure 3:**
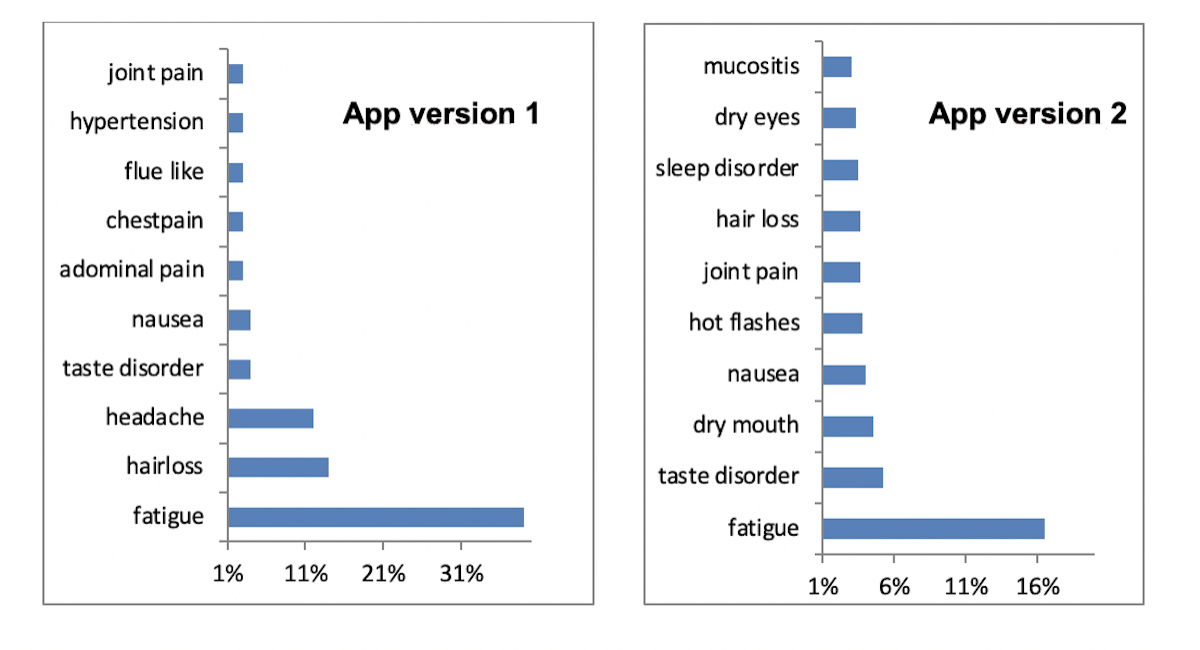
Most frequently reported side-effects in both app versions.

The most commonly reported symptom in both groups was fatigue, although this was indicated twice as often (37% vs 18%) in the group of early stage breast cancer (version 1 of the app). This slightly younger and supposedly more fit patient group also frequently reported symptoms, including hair loss, headache, taste disorder, nausea, and abdominal pain (Figure 3), while users of version 2 of the app frequently reported symptoms, including taste disorder, dry mouth, nausea, hot flashes, and joint pain. Unfortunately, owing to the heterogeneity of drugs and limited information on dosage, we were not able to analyze potential associations of symptoms with the applied treatment regimens and settings (eg, adjuvant vs noncurative).

### Utility of the Smartphone App Versions

Questionnaires from all patients included in the prospective trial were available for rating version 1 of the app, and questionnaires from 67 patients were available for rating version 2 of the app. No patient died or was censored from analysis. Overall satisfaction with both app versions was high. In our experience, the vast majority of patients were able to use both app versions intuitively to report their symptoms, although a few elderly individuals and nonapp users in particular required instructions from a nurse or physician. According to the answers received from the patient questionnaires (Table 2), both app versions were rated helpful, although version 2 of the app was a clear favorite among users (*P*=.003). Patients in both groups also stated that the app had a positive effect on their doctor visits and that the symptoms were encountered for shared review. Importantly, nearly all patients felt reassured that their personal data were treated confidentially and stated that they would recommend the easy-to-use Consilium Care app version 2 to other patients (Table 2). Although not statistically significant, version 2 of the app appeared to be more helpful for dealing with the symptoms of illness (*P*=.057). Of note, no technical issues or data safety concerns were raised during the course of this study.

**Table 2:** Comparison of the usability of the two smartphone app versions.

Statement	Good and very good agreement with the statement, % of patients		*P* value
	Version 1 (n=86)	Version 2 (n=67)	
I find the app helpful	62 (72%)	61 (91%)	.003
The app is easy to use	N/A^a^	66 (99%)	N/A^a^
The app helps me deal with the symptoms of my illness	53 (62%)	51 (76%)	.057
The app has had a positive effect on doctor visits	69 (80%)	54 (81%)	.96
My records were taken into account by the doctor during consultations	81 (94%)	60 (90%)	.29
My symptoms are taken seriously by the doctor	84 (98%)	65 (97%)	1.0
I believe that my personal data will be treated confidentially	84 (98%)	67 (100%)	.50
I would recommend the app to other patients	84 (98%)	65 (97%)	1.0

^a^N/A: not applicable.

## Discussion

In this article, we demonstrated that collaborative patient-doctor symptom review was likely to affect the number of digital symptom data entries. This finding adds to the increasing published data on the effects of electronic symptom reporting of patients undergoing systemic anticancer therapy [2-7]. Several studies have also reported high compliance rates with patient-reported outcomes in oncology trials, improved accuracy, and completeness of data, as well as positive effects during routine cancer treatment [3-5]. Recent findings in breast and prostate cancer patients undergoing radiotherapy, however, indicated that the acceptance and possible benefits of a mobile app might be higher in younger or less fit patients [14]. Likewise, in our study, the recording of well-being during systemic treatment seemed to be more important to the patient group with presumably advanced cancer stages and less fit patients, who needed and expected improved disease control.

The high amount of data entries and answers from the patient questionnaires (Table 2) suggested a considerable ease of use of the Consilium Care app associated with possible benefits for self-empowerment. As a result of inputs from doctors, nurses, and patients at different ages and cancer entities for the development process of version 2 of the app, we designed and implemented automatic reminders (push notifications) and refined the operation for the structured assessment of side-effects and quality of life, as well as symptom history charts, as these are important functions of a mobile app [4,15,16].

Personal communication from patients further indicated a great interest in alert functions, which should be displayed based on data input, and an intrinsic willingness to share this information with the treatment team. Although several patients and doctors showed interest in entering vital data (eg, glucose, blood pressure, and weight), this feature has not yet been implemented in either app version. Previously, in version 1 of the app, we had shown that the use of the Consilium Care app had the potential to stabilize the daily functional activity of cancer patients and that more distinct symptom entries were received from those users who shared reporting with their doctors [1]. Patients using version 2 of the app obviously indicated higher amounts of distinct symptoms (*P*=.038; Table 1) and indeed reported on their well-being every single day. Therefore, it is likely that the implementation of a request for shared symptom review, as integrated in version 2 of the app, might have positively affected the frequency and number of digital symptom data entries [15,17]. However, owing to several limitations of this study, the treatment context, applied medication schedules, and differences in the number of available symptoms could not be evaluated in more detail for the interpretation of symptom entries.

Since almost all patients stated that they would recommend the app to other patients (Table 2), the general aspects of usability obviously did not negatively affect the patient rating of either app version. Our assumption that patients would positively encounter the intended “fresh look” of the interface and design in version 2 of the app, however, was currently not tested in a specific questionnaire and thus cannot be attributed to an increased number of symptom data entries [4]. In an ongoing observational study, we are investigating how far collaborative symptom reviews might affect both the quality and severity grading of symptoms, as well as reliability of data entries in association with patient outcomes [13]. However, in general, patients rated version 2 of the app as being more helpful, although a considerable benefit in dealing specifically with the symptoms of illness was not demonstrated (Table 2).

As mentioned, neither version of the app was equipped to send questionnaires, and calls from nurse specialists were not intended. We can only speculate regarding how far occasional push notifications, ample choice of symptoms, and listing of frequently selected “favorites” or “last used” symptoms might have influenced patients’ motivation and input selection, as unfortunately, we did not include these variables in our end-of-study assessment [18,19]. However, the increasingly careful symptom recording provided by ePROs, along with improved symptom management in routine outpatient care, demonstrated a reduction in both unplanned hospitalizations and disease burden [2]. In their study, Basch et al [2] asked patients to report (between regular visits and upon weekly email prompts) on 12 common symptoms available for grading on a 5-point scale from 0 (not present) to 4 (disabling) based on clinical criteria (CTCAE). Data from 441 patients contributed to a total of 84,212 individually reported symptoms during a mean period of 7.4 months. Of note, when considering these numbers, a total of more than 250,000 symptom entries would have resulted from our patient cohort, although it cannot be ruled out that patient motivation for reporting may decrease over time. Due to the descriptive characteristic of our study, we were unable to determine a definite pattern in symptom recording with respect to the duration of the observational period (6 vs 12 weeks). On a personal communication level, most of the physicians who explored the Consilium Care app confirmed that, in particular, the summative picture of a timeline history for symptoms could provide more information than a thousand numbers [4]. Most of the patients also indicated that their use of the app had a positive effect on doctor visits with a focus on the evaluation of symptoms.

As patients frequently reported cognitive impairments, the diary characteristic of apps in general might appear helpful to frequently capture and recall disease-related information [17]. Interestingly, users of version 1 of the app indicated their fatigue on almost 6 of 7 days per week, potentially related to a pronounced effect of menopausal symptoms after chemotherapy, while users of version 2 of the app, who had a presumably more severe disease course, indicated their well-being every single day, a finding that could be associated with a lack of wording for cognitive needs in the available CTCAE [17].

In summary, recent published data indicate that efforts in patient-centered design and usability of mobile apps could contribute to the essential collection and communication of high-quality patient-reported outcome data for the timely management of treatment-related side-effects and toxicities [18]. There is a need to further explore how far the range of available symptoms or the intention for shared symptom review may affect the frequency or number of reliable data entries. In the context of increasingly complex cancer therapies, the growing use of oral anticancer drugs, and COVID-19–related efforts to provide remote care, implementation strategies for patient communication and adherence [19] should be iteratively challenged in clinical practice.

## Abbreviations

CTCAE: Common Terminology Criteria for Adverse Events
ePRO: electronic patient-reported outcome
